# A protocol to evaluate the impact of embedding Public and Patient Involvement in a structured PhD program for stroke care

**DOI:** 10.3389/fresc.2022.877598

**Published:** 2022-07-22

**Authors:** Frances Horgan, Olive Lennon, Anne Hickey, Jan Sorensen, Thilo Kroll, Deirdre McCartan, Patricia Hall, Geraldine O'Callaghan, Clare Fitzgerald, Julianne Hickey, Martin Fahy, Philip Osborne, Mary Scullion, Clíodhna Ní Bhroin, David J. Williams, The iPASTAR Collaborative Doctoral Award Programme Team

**Affiliations:** ^1^Improving Pathways for Acute Stroke and Rehabilitation (iPASTAR) Collaborative Doctoral Award Programme, Division of Population Health Sciences, Royal College of Surgeons in Ireland (RCSI) University of Medicine and Health Sciences, Dublin, Ireland; ^2^School of Physiotherapy, Royal College of Surgeons in Ireland (RCSI) University of Medicine and Health Sciences, Dublin, Ireland; ^3^School of Public Health, Physiotherapy and Sports Science, University College Dublin, Dublin, Ireland; ^4^Department of Psychology, Royal College of Surgeons in Ireland (RCSI) University of Medicine and Health Sciences, Dublin, Ireland; ^5^Healthcare Outcomes Research Centre, Royal College of Surgeons in Ireland (RCSI) University of Medicine and Health Sciences, Dublin, Ireland; ^6^University College Dublin (UCD) Centre for Interdisciplinary Research, Education and Innovation in Health Systems, Dublin, Ireland; ^7^Health Research Board (HRB), Public and Patient Involvement (PPI)-Ignite University College Dublin, Dublin, Ireland; ^8^Improving Pathways for Acute Stroke and Rehabilitation (iPASTAR) Public and Patient Involvement (PPI) Champion iPASTAR Collaborative Doctoral Award Programme, Dublin, Ireland; ^9^School of Medicine Royal College of Surgeons in Ireland (RCSI) University of Medicine and Health Sciences, Dublin, Ireland; ^10^Department of Geriatric and Stroke Medicine, Beaumont Hospital, Dublin, Ireland; ^11^Improving Pathways for Acute Stroke and Rehabilitation (iPASTAR) Collaborative Doctoral Award Programme, Royal College of Surgeons in Ireland (RCSI) University of Medicine and Health Sciences, Dublin, Ireland

**Keywords:** stroke care, public patient involvement (PPI), engagement (involvement), evaluation, structured PhD programme

## Abstract

**Background:**

Embedding Public and Patient Involvement (PPI) in postgraduate research has been recognized as an important component of post-graduate training, providing research scholars with an awareness and a skillset in an area which prepares them for future roles as healthcare researchers. Improving Pathways for Acute STroke And Rehabilitation (iPASTAR) is a structured PhD training program [Collaborative Doctoral Award (CDA)] which aims to design a person-centered stroke pathway throughout the trajectory of stroke care, to optimize post-stroke health and wellbeing. PPI is embedded at all stages.

**Purpose:**

The iPASTAR research programme was strongly informed by a round-table PPI consultation process with individuals who experienced stroke and who provided broad representation across ages, gender, geographical locations (urban and rural) and the PhD themed areas of acute care, early supported discharge and lifestyle-based interventions after stroke. Four PhD scholars taking part in the CDA-iPASTAR now work collaboratively with four stroke champions, supported by a wider PPI advisory panel.

**Methods:**

This study will evaluate the process and impact of embedding PPI during a PhD program. We will conduct a longitudinal mixed-methods evaluation, conducting focus groups at 24, 36, and 48 months to explore the experiences of the key stakeholders involved. The participants will include PhD scholars, PPI partners (PPI Advisory Group and PPI Champions), PhD supervisors and a PPI manager. An independent researcher will conduct the evaluation. We will include focus groups, individual interviews and participant reflections. Qualitative data will be analyzed using thematic and content analysis, quantitative data will be analyzed using descriptive statistics.

**Discussion:**

PPI and patient voice initiatives bring together researchers, family, and people with health care issues into meaningful dialogue and allow the development of a patient-voice learning network. Embedding PPI training within a PhD program can build meaningful capacity in PPI partnerships in stroke research.

## Introduction

Internationally, stroke is a major cause of death, and the commonest cause of adult-acquired disability The European Burden of Stroke Report indicates that between 2015 and 2035, there will be a 34% increase in total stroke events in the European Union from 613,148 to 819,771 (1). There are ~11,000 strokes/Transient Ischaemic Attacks (TIA) in Ireland annually. This number is projected to rise by 50% over the next 8 years as the population ages. The average age for first stroke is 74-years, with the EU Burden of Stroke Report approximating 8,000 additional new stroke cases every year in Ireland (2).

The European Stroke Organisation (ESO), Stroke Alliance For Europe (SAFE), European Society of Minimally Invasive Neurological Therapy and the European Academy of Neurology in 2019 jointly called for urgent, acute and longer-term strategies to address this growing demand on stroke services and identified that multifaceted approaches are required to turn the stroke burden around (3). In Ireland, the recent Irish national audit of stroke (INAS) (4) highlighted that stroke care in Ireland is fragmented. While improvements have been made in acute stroke unit availability and access to thrombolysis, this disjointed approach to care fails to provide an effective, integrated, high quality system for stroke prevention, treatment and rehabilitation (4). The European SAFE report (5) recommendations for coherent systems in acute stroke care, includes clear protocols and processes of appropriate emergency pre-hospital and within hospital emergency care and for a comprehensive system of rehabilitation appropriate to and tailored for individual patient goals and stroke prevention which have yet to be implemented. The recent Stroke Action Plan for Europe (SAP-E) sets targets for the implementation of evidence-based preventive actions and stroke services to 2030 (6). Stroke leads to a “disruption of life, for patients and their carers who engage in a process of adapting and rebuilding a post-stroke life and identity”. They value information that helps them “prepare for and adjust to this new situation and optimal rehabilitation is a main concern and goal for patients and carers” (7).

INVOLVE defines public and patient involvement (PPI) in research as “research carried out “with” or “by” members of the public rather than “to”, “for” or ‘about' them”^1^. PPI in health research can have an impact on research quality, relevance, impact and integrity (8–10). PPI in research therefore involves an active partnership between members of the public and researchers. This means that members of the public work alongside the research team and are actively involved in contributing to the research process as advisers and possibly as co-researchers (11). PPI has been shown to have a positive impact on health and social care research (12). Dawson et al. (13) described three main reasons for PPI in health services research; normative or emancipatory, consequentialist or efficiency-oriented and political and practical. Normative or emancipatory reasons assert that patients have a right to be involved in research that might affect them and reduce power imbalances between researchers and PPI contributors (14–16). Consequentialist or efficiency-oriented reasons (15, 17) bring a “lived experience and real-world perspective which contributes to improving the efficiency and value of research through various mechanisms” (18). Lastly, the rationale for “political or practical grounds can lead to co-construction of knowledge through alliances between researchers and patients”, which can increase the transparency of research (18–20).

Researchers and clinicians may not always have first-hand experience of an illness, disease or service that they wish to research. “Patient voice” is a term that has become frequently used in health and social care settings and is often used to describe a compilation of many patients' and carers' expressed feelings, concerns, and experiences during an illness (21, 22). PPI representatives can provide researchers with insights into what it is like to live with a particular illness, and what it is like to be a service user of a treatment or health service thus helping to make health service research more relevant to the needs of patients, carers and service users. INVOLVE^1^ and the National Institute for Health Research (NIHR) (11) describe how patients and the public can become involved in all stages of the research process including: “prioritization of studies, design and management of studies, data collection and analysis, dissemination and reporting of findings”^1^ (11). INVOLVE^1^ has developed guidelines on co-producing a research project, an approach in which researchers, practitioners and the public work together, “sharing power and responsibility from the start to the end of the project, including the generation of new knowledge”. This guidance can help both researchers and members of the public to have clarity about what is meant by co-producing a research project^1^.

**Figure 1:**
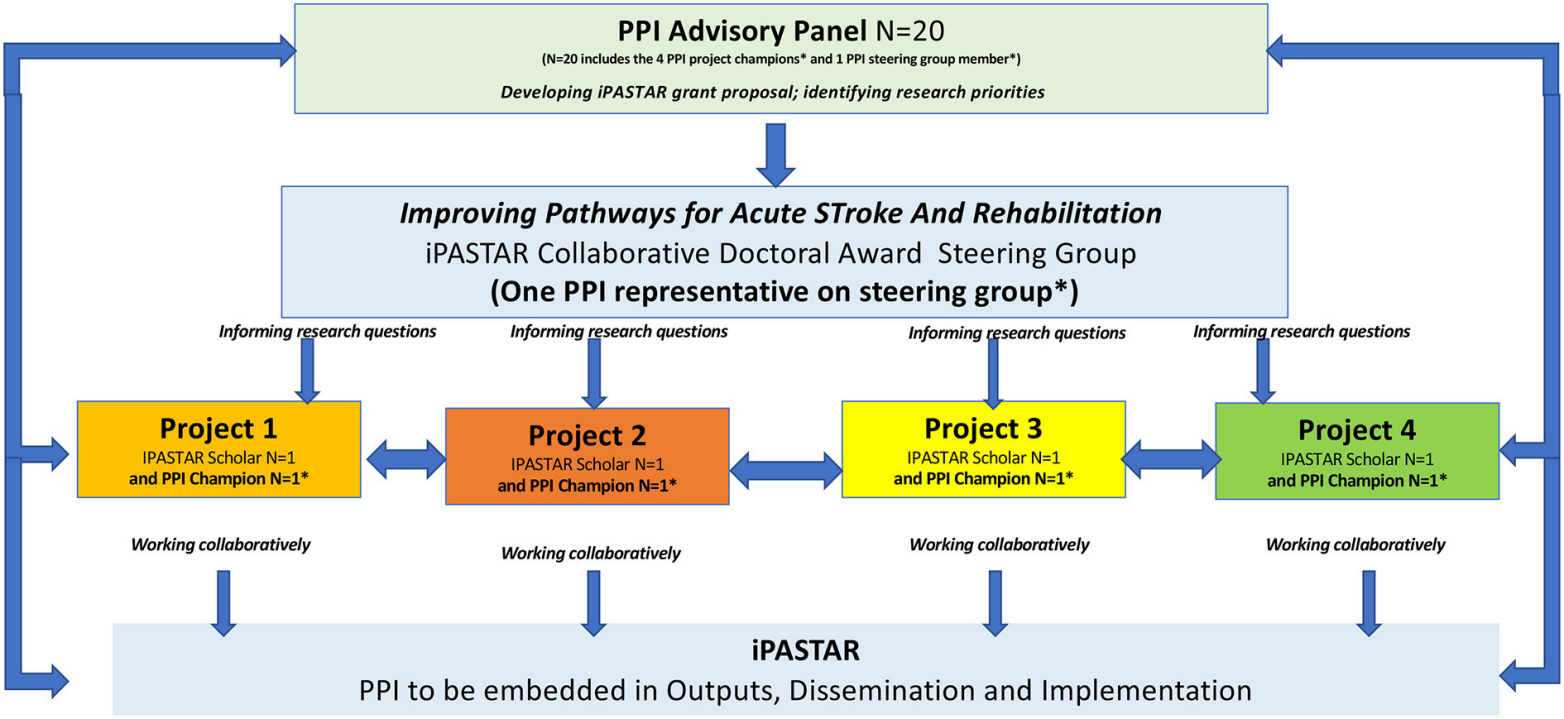
Overview of PPI in the iPASTAR CDA programme.

PPI is gaining momentum in Irish research and is of increasing relevance to many health research stakeholders [Irish Platform for Patient Organisations, Science and Industry (IPPOSI)] (23). The Health Research Board of Ireland (HRB) developed an implementation plan to support PPI both within the HRB and through HRB-supported projects and programs. They were the first funding agency in Ireland to do so (24). In 2021, the Health Research Board (HRB) launched and funded PPI-IGNITE-II to support research institutions develop a national network to advance the involvement of the public, patients and carers in health and social care research, from the generation of ideas to delivery of results ^2^. This initiative, in collaboration with the Irish Research Council (IRC), will see the development of the National Network of PPI centers across seven universities and over 80 local, national and international partners who will work together to advance PPI on the island of Ireland and beyond. The HRB Collaborative Doctoral Awards in Patient-focused Research (CDA) scheme, launched in 2017, aims “to support excellent doctoral training programs for a cohort of individuals including those from academic health-related disciplines and particularly those from health and care practice, in the conduct of patient-focused research”. One such Health Research Board (HRB) Collaborative Doctoral Award (CDA) programme, in stroke care (iPASTAR), commenced in 2020 ^3^. The iPASTAR-Improving Pathways for Acute STroke And Rehabilitation programme “will generate a cohort of post-doctoral researchers with transferrable skills who can make significant future impact in stroke care with necessary expertise in the generation of research evidence to support cost-effective management of stroke care”^3^. PPI has been embedded in iPASTAR in a number of areas, including the grant writing stage and in governance structures (advisory panel, management group), with each of the PhD projects having a PPI PhD project champion who will play a key role in dissemination activities, as summarized in Figure 1.

There are very few examples describing how to operationalize PPI in doctoral training programs. Tomlinson et al. (12) described four case studies demonstrating how PPI could be incorporated at various stages during doctoral research. They described the approaches used by four doctoral researchers to incorporate PPI in their research studies from study design to the dissemination of findings and recommend greater uptake of PPI in doctoral research with adequate support (12). Foley et al. (25) described a protocol to evaluate the learnings from embedding formal and experiential PPI training and education across a PhD program in multimorbidity. This work remains in progress. While there is a pressing need to gather PPI perspectives and evaluate PPI in doctoral research in general, the impact and burden of stroke on the individual and family could potentially limit PPI activity in stroke specific research (26). No guidance on how best to include PPI in stroke research (including at doctoral level training) currently exists (27). This proposed study aims to evaluate the process of embedding PPI during a structured PhD program in stroke care. The opinions and experiences of PPI participants, doctoral students and academic supervisors involved in the process will be evaluated.

## Materials and analysis

### Study design

We will conduct a longitudinal descriptive qualitative exploratory study. The study will be conducted in accordance with the Consolidated Criteria for Reporting Qualitative Research guidelines (COREQ) (28).

The objectives of the study are:

- To explore the experiences and perspectives of PPI stakeholders (PPI partners, PhD students, PhD supervisors and PPI managers), participating in a stroke doctoral program;
- To explore the process of embedding PPI in a stroke doctoral program from the perspective of PPI partners, PhD students, PhD supervisors and PPI managers;
- To evaluate the impact of embedding PPI in the iPASTAR stroke doctoral programme, and the four doctoral projects in relation to the research and research process.

### Participants and recruitment

The sampling frame will include the following:

- 20 PPI advisory group members/partners (four of whom are PPI champions, one of whom is a member of the steering group);
- four PhD scholars;
- 8 PhD supervisors:
- a PPI representative from a national stroke advocacy organization (*n* = 1) and
- the University PPI programme manager (*n* = 1).

The potential sample of all identified stakeholders is *n* = 34. To maintain independence and objectivity, an experienced and independent qualitative researcher, not associated with the iPASTAR programme will conduct the focus groups and interviews. The authors will be potential participants in the study. Potential participants will be invited to take part in the study through a gatekeeper, who will outline the details of the study, and with consent share contact details with the independent researcher who will organize the process of informed consent and scheduling of interviews.

### Evaluating impact

Qualitative interviews will be conducted to understand the impact of the various PPI activities in iPASTAR (Figure 1 overview of PPI in the iPASTAR CDA), how PPI was established, how it operates and to explore the experiences and views of the PPI partners (what did we do? what was discussed? what did we change? what was the impact? The impact of PPI on the research and research process, analysis and writeup, dissemination), PhD scholars and PhD supervisors on embedding PPI in a structured stroke care PhD program. The experiences and views of these key stakeholders will be explored using a combination of focus groups and individual interviews conducted at specific time points throughout the program.

### Data collection

Focus groups and interviews will be semi-structured and will be guided by an interview topic schedule informed by current literature and with input from the PPI advisory group, for example to examine composition, power and influence of the group, fluctuation of participants.

The discussion groups and interviews may take place remotely or in person and will be conducted at 24, 36, and 48 months. The iPASTAR programme duration is 60 months (Table 1). Discussion groups and interviews will be audio-recorded. Three focus groups will be conducted with PPI partners, supervisors, managers (24, 36, and 48 months) and individual interviews will also be conducted with PhD scholars (24, 36, and 48 months). In evaluating the PPI process, we will explore ways of working, communication, roles and responsibilities, documents (terms of reference), PPI at different levels (iPASTAR and PhD study level), agenda setting and mutual engagement and inclusiveness.

**Table 1:** Overview of methods, the evaluation process.

	**1. To explore the experiences and perspectives of PPI stakeholders (PPI partners, PhD students, PhD supervisors and PPI managers), participating in a stroke doctoral program;**	**2. To explore the process of embedding PPI in a stroke doctoral program from the perspective of PPI partners, PhD students, PhD supervisors and PPI managers;**	**3. To evaluate the impact of embedding PPI in the iPASTAR stroke doctoral programme, and the four doctoral projects in relation to the research and research process**.	**Data Analysis**
PPI Panel/champions (*n* = 20)	Focus groups or Semi-structured interviews at 24, 36, and 48 months	Focus groups or Semi-structured interviews at 24, 36, and 48 months	Focus groups or Semi-structured interviews at 24, 36, and 48 months	Thematic analysis
PhD scholars (*n* = 4)	Focus group at 24, 36, and 48 months and Semi-structured interviews (SSI)	Focus group at 24, 36, and 48 months and Semi-structured interviews and Documentary analysis and Notes from group reflections months 24, and 48	Focus group at 24, 36, and 48 months and Semi-structured interviews	Thematic analysis Content analysis
PhD supervisors (*n* = 8)	Focus group at 24, 36, and 48 months	Focus group at 24, 36, and 48 months and Documentary analysis	Focus group at 24, 36, and 48 months	Thematic analysis
PPI managers (*n* = 2)	Focus group or Semi-structured interviews at 24, 36, and 48 months	Focus group or Semi-structured interviews at 24, 36, and 48 months	Focus group or Semi-structured interviews at 24, 36, and 48 months	Thematic analysis

We will include a documentary analysis (29) for example what information is shared in written form at iPASTAR and scholar level; meeting agendas etc. This qualitative approach offers the opportunity to gain an insight into the experiences and perspectives of the key PPI stakeholders in a structured PhD program. This approach will also allow the independent researcher to be flexible and adapt interview questions in response to participants.

In addition, we will ask the four PhD scholars to reflect on their experience of embedding PPI in their PhD projects using a self-facilitated reflection followed by a group reflection. These reflections will be guided by the Gibbs Reflective Cycle (30), to provide structure to the learning from their experience and ongoing and future learning and development. The scholars will retain a written record of a group reflection. These reflections may also include details to reflect on the time spent in PPI activities and planning, for example preparing and facilitating meetings (e.g., possible self-reflective questions, what did we do, discuss and change, and what was the impact of this?).

### Data analysis

All transcripts will be analyzed using a reflexive approach to thematic analysis which followed the six-phase guide provided by Braun and Clarke to identify themes within the data (31, 32). Interview transcripts will be read in their entirety by an independent qualitative researcher. Following this, inductive coding will commence, systematically highlighting segments of data which include words and phrases relevant to each code (33). Codes with similar meaning will be grouped and collapsed to form themes pertinent to experiences, perceptions of enablers and barriers to embedding PPI. The themes will be reviewed to discuss over-arching themes and sub-themes in the context of accurately reflecting the supporting data.

### Ethical considerations

The study will be submitted for ethical approval to the Research Ethics Committee of the RCSI University of Medicine and Health Sciences. Written informed consent will be obtained from all participants to include publication of the anonymized responses. Transcripts will be shared with study participants to ensure member checking before the analysis is finalized. All participants will be described using coded identification numbers. The involvement of an independent qualitative researcher who is responsible for managing and analyzing the qualitative data will mitigate any potential risks associate with the source of the data. A particular risk associated with the study is the small pool of study participants and that they are known to one another in their different roles as PPI advisory panel member, PPI champion, PhD scholar and PhD supervisor There are established relationships between PhD scholar and supervisor and PPI champion and PhD scholar. This could be interpreted as a power imbalance or dependent and unequal relationship. By involving an independent researcher, not known to the study participants and external to the involved institutions in this research consortium, we hope this will facilitate an open and honest discussion of their experience of PPI in a safe environment. The PPI partners will receive clinical support from the academic institution in light of any potential risk of role conflation/perceived risks to them as patients if they had anything critical to share. We do not anticipate any additional risks to the participants from participating in the study.

### Dissemination of findings

We will publish the findings in a peer review journal and apply the Guidance for Reporting Involvement of Patients and the Public (GRIPP-2) tool (34). Our PPI partners (PPI advisory group and PPI champions) will be involved as co-authors and as presenters alongside the researchers at different events in the dissemination of the study findings to people living with stroke, other PPI stakeholders and stroke researchers.

## Discussion

Revisiting Dawson et al.'s (13) discussion around the main reasons for PPI, the normative or emancipatory approach, describes how patients have a right to have an input to research on their condition and that this can reduce power imbalances between researchers and patients. Dawson's description of the PPI process was retrospective and included PPI contributors and the researcher. We will also explore the perspectives of supervisors and advocacy/PPI managers specific to stroke care pathway research. Looking at the consequentialist or efficiency-oriented approach, allows PPI to create a real-world and lived-experience perspective and improves the value of research through a number of possible mechanisms. It also increases the relevance of the research to patients, positively influences recruitment and retention rates of study participants; and expands the dissemination of findings. Gibson et al. (35) explored the theoretical directions for an emancipatory concept of PPI, and a four dimensional framework for analyzing the nature of PPI, which provides the “co-ordinates along which new ‘knowledge spaces' for PPI could be constructed” which facilitates and supports the emergence of social networks of knowledgeable actors capable of engaging with professionals'. PPI and patient voice initiatives bring researchers, family, and people with health care issues into meaningful dialogue and allows the development of a patient-voice learning network. PPI is also relevant in the context of coproduction in healthcare which described how patients contribute to the provision of health services as partners of professional providers. Co-production is receiving increasing attention, although insights into the processes involved is limited (36, 37).

Embedding PPI training within a PhD program is a novel approach that requires critical examination to guide future educational and research practices in doctoral studies. PPI in stroke research and in the context of a collaborative doctoral program may present additional challenges that warrant consideration to guide future stroke related research. As the focus of the iPASTAR CDA is the design of a person-centered stroke pathway to optimize health and wellbeing, PPI is embedded in this CDA application at all stages. Our proposed study aims to evaluate the process and impact of embedding PPI in a structured doctoral programme in stroke care.

## Plain English summary

### Why are we doing this research?

Public and patient involvement (PPI) can provide researchers with insights into what it is like to live with a particular illness, and what it is like to be a service user of a health service. PPI is new for many PhD students for which there is little guidance and training. We plan to explore the experience of PPI partners living with stroke and researchers involved in a structured PhD program in stroke care.

### What is this study trying to find out?

We wish to determine how a team of stroke researchers and PhD students, who worked with public and patient partners in their research projects to develop a person-centered stroke pathway, learned from this process, the impact it had and what people living with stroke and their families/carers thought about their involvement.

### How will we do this research?

A panel of PPI partners, PhD scholars and their academic supervisors will be invited to take part in a focus group or interview at the middle and late stages of the stroke training program. During these discussions we will explore their experiences and will analyze the results to understand stakeholders' experiences of PPI in active research and how these experiences influenced the research process, and what everyone gained and learned from this experience.

### What will we do with the results of this study?

We will discuss and develop with our PPI partners different methods of sharing the results with different audiences and will invite our PPI members to share their experience of being involved in a stroke doctoral research program and what they learned during this process. This will allow us to develop guidelines on how PPI can be integrated into health services research and guide researchers to what worked well and what might need further refinements and thoughts. We hope that this will encourage others to get involved in research in the future. This would allow PPI partners to have their voice heard in relation to topics that they would like to see researchers work on with them and building PPI partnerships in research.

## Data availability statement

The original contributions presented in the study are included in the article/supplementary material, further inquiries can be directed to the corresponding author.

## Author contributions

All authors contributed to the development of the manuscript and approved the protocol paper prior to submission.

## Funding

This study was supported by a Health Research Board of Ireland (HRB) Collaborative Doctoral Award (CDA) reference CDA-2019-004.

## Conflict of interest

The authors declare that the research was conducted in the absence of any commercial or financial relationships that could be construed as a potential conflict of interest.

## Publisher's note

All claims expressed in this article are solely those of the authors and do not necessarily represent those of their affiliated organizations, or those of the publisher, the editors and the reviewers. Any product that may be evaluated in this article, or claim that may be made by its manufacturer, is not guaranteed or endorsed by the publisher.

## Footnotes

^1^ https://oxfordbrc.nihr.ac.uk/ppi/ppi-researcher-guidance/what-is-patientand-public-involvement

^2^https://ppinetwork.ie/.

^3^https://ipastar.eu.
